# An Alternative Paradigm for the Role of Antimalarial Plants in Africa

**DOI:** 10.1100/2012/978913

**Published:** 2012-04-19

**Authors:** Steven Maranz

**Affiliations:** David H. Murdock Research Institute, Kannapolis, NC 28081, USA

## Abstract

Most investigations into the antimalarial activity of African plants are centered on finding an indigenous equivalent to artemisinin, the compound from which current frontline antimalarial drugs are synthesized. As a consequence, the standard practice in ethnopharmacological research is to use *in vitro* assays to identify compounds that inhibit parasites at nanomolar concentrations. This approach fails to take into consideration the high probability of acquisition of resistance to parasiticidal compounds since parasite populations are placed under direct selection for genetic that confers a survival advantage. Bearing in mind Africa's long exposure to malaria and extensive ethnobotanical experimentation with both therapies and diet, it is more likely that compounds not readily overcome by *Plasmodium* parasites would have been retained in the pharmacopeia and cuisine. Such compounds are characterized by acting primarily on the host rather than directly targeting the parasite and thus cannot be adequately explored *in vitro*. If Africa's long history with malaria has in fact produced effective plant therapies, their scientific elucidation will require a major emphasis on *in vivo* investigation.

## 1. Introduction

In popular African thought, there is a conviction that the continent's vast traditional pharmacopeia includes potent indigenous therapies that can outperform medicines from industrialized countries [1–3]. This perception appears to be contradicted by both the historical high burden of malaria in sub-Saharan Africa and the increasing role played by foreign medical intervention in Africa's health crises [4]. With respect to malaria, the current success of artemisinin combination therapy in reducing transmission rates across the continent is not matched by results from a comparable indigenous counterpart [2, 4]. If an African plant therapy with artemisinin-like epidemiological effects exists, it is either not currently in widespread use, or its effects are flying under the radar. The counter argument often heard in Africa is that although highly effective cures do exist, they constitute hidden knowledge, with the secret formulas held by a few old men, who will not even on their deathbeds transmit the information to their own sons [5]. If this is true, then the tradition of secrecy serves no public health purpose, nor does it have any future other than extinction.

 The narrative of a hidden indigenous cure can be seen as a response to the success of modern pharmaceutical drugs, especially in treating severe childhood malaria. To most observers, the sharp reduction in child mortality rates in localities where modern treatment is accessible dramatically emphasizes the performance gap between traditional plant therapies and packaged tablets obtained in clinics and pharmacies [2, 4]. Since Africa has a very rich medicinal plant tradition, the apparent failure to treat the millennia-old scourge of malaria demands an answer or an alibi, which the stories of secrecy provide. This response to a sense of failure concedes a frame-of-reference advantage to Western medicine, which perceives any malaria infection as unacceptable, thus requiring therapies that dramatically change the status quo [4, 6].

 However, when a longer-term view is taken, Africa's lack of an indigenous wonder drug has a compelling epidemiological basis. Drugs such as chloroquine were very effective over a period of decades, but eventually failed. Artemisinin derivatives are currently very effective, but reports of an incremental creep in resistance are emerging in many places [7, 8]. If Africa had in its past indigenous equivalents to chloroquine or artemisinin that had entered widespread use, they would in all likelihood have failed as well. Given Africa's long exposure to malaria, it is much more logical to consider the possibility that traditional African therapies focused on treatments not easily overcome by *Plasmodium* parasites. Identifying such therapies requires understanding the differing goals of the conventional pharmaceutical model compared to the African therapeutic context.

 Some herbal medicine advocates argue that whole plant extracts are inherently combination therapies, supplying several different antimalarial compounds at once, making traditional plant therapy incapable of driving acquisition of resistance. Historical data to evaluate such a claim are lacking: for instance, even though the use of *Artemisia annua* to treat fever and chills in China was recorded almost 1,700 years ago by Ge Hong [9], we have no way of knowing if the treatment was either widely known or commonly used against malaria. There is neither a historical record of frequency and intensity of *A. annua* use nor a contemporary study measuring resistance to artemisinin in an area of current ethnopharmacological use of *Artemisia*. What we do know is that potent inhibitory compounds put parasites under strong selection pressure that rewards parasite genetic variations that confer a survival advantage. A fast-acting compound like artemisinin can put blood stage parasites through a selective bottleneck before accompanying herbal compounds have an impact, potentially negating the combination therapy value. Additionally, in a weak or dilute traditional extract, a compound that inhibits at nanomolar concentration can still be active, thus selecting for resistant parasites, while the less potent accompanying compounds remain below their activity threshold—in effect functioning as a monotherapy. The point here is that there may be good epidemiological reasons for why the search for antimalarial compounds with low nanomolar efficacy has not yet identified a potent antiplasmodial plant like *A. annua* in the African pharmacopeia.

### 1.1. Conventional Antimalarial Drug Research

Despite the aura of secrecy in African ethnomedicine, a considerable catalog of plant species and medicinal applications has emerged over time, providing an extensive inventory of candidates for evaluation [10, 11]. Most contemporary research into African antimalarial plants has been directed towards finding an African equivalent to quinine or artemisinin and is based almost entirely on *in vitro* screening [12, 13]. A study of Kenyan antimalarial plants concluded that the majority of extracts had low or no activity, with a best IC_50_ value of 3.65 *μ*g/mL [14]. A South African review of 220 antimalarial plant species identified just six compounds from *in vitro* screenings with IC_50_ values below 1 *μ*g/mL and selectivity indexes greater than 10 (ten times more inhibition of *P. falciparum* than of mammalian cell lines) [15]. The most potent antiplasmodial compound identified had an IC_50_ of 240 ng/mL, which is approximately 10-fold weaker than artemisinin. A similar review of 109 West African plant species identified one compound, gedunin, with IC_50_ values comparable to artemisinin [16]. Gedunin is a limonoid tetraterpene from the mahogany family, the members of which are widely used for treating malaria [11, 17]. Although the *in vitro* selectivity index of gedunin against mammalian cells was favorable [17], extracts from plants in this family have been found *in vivo* to act as abortifacients [18, 19]. Toxicity issues are a central concern in IC_50_ studies geared towards identifying compounds with high biological activity at very low doses [15, 20]. However, the large majority of African plants and extract fractions evaluated have not killed parasites at submicromolar doses. To date, no new commercial malaria drug has emerged from *in vitro* screening of African plants.

 With recent advances in molecular biology and genomics, many malaria drug research programs have adopted a high tech strategy of identifying a key parasite metabolic or transcriptional pathway, then developing a suitable assay for measuring inhibition of the pathway, followed by screening a vast library of compounds for hits [21–23]. The compound libraries generally consist of pure compounds that may be either synthetic or plant derived [24]. Hits are scored on the basis of inhibition versus concentration: the top performers or those exhibiting IC_50_ values below a specified threshold are then screened for mammalian cell toxicity [25]. A final short list of compounds is tested *in vitro* against parasites to verify activity against live pathogens [26]. Those that do successfully inhibit parasites are examined in more detail to verify disruption of the metabolic target. Compounds emerging from this process may finally be tested *in vivo* using a mouse malaria model [27]. The chance of failure during first *in vivo *use of a drug selected from a blind screening assay is high: in the absence of a history of mammalian use, problems at multiple levels are likely to occur. While technical issues such drug absorption, drug stability, and metabolism can be tweaked through additional synthetic manipulation of the identified compound, they can still present a significant hurdle. Since the molecular structures of the compounds in the library are known in advance, computer-aided comparisons of structures can pinpoint structure-activity relationships [25]. This information in turn can guide synthesis of analogs that are potentially more efficacious, more stable, and more readily absorbed *in vivo* [23]. Modified lead compounds that are successful in this regard still face the considerable challenge of producing the desired antiparasitic effect in a human host without also generating unintended consequences. Organisms share a vast number of the same or similar metabolic and genetic features, so that a drug acting as desired on a parasite may yet have unforeseen interactions with related pathways in the host.

 Despite the technical tour-de-force of the high throughput screening methodology, it is intrinsically a blind process, in which the ultimate goal of *in vivo* efficacy is the very last step to be evaluated. This process is exactly the inverse of an approach based on African traditional medicine, where some kind of *in vivo *efficacy is presumed from common use, while the mode of action is unknown. It should not be forgotten that human populations in Africa have been intensively exposed to malaria over many thousands of years—thus, a comprehensive high throughput screening of plants has already occurred, with all the assays done *in vivo* with human subjects. The contrast between the empirical, *in vivo* traditional approach in endemic malaria areas and the random, high throughput, theoretical approach—usually conducted in laboratories far from the tropics—could hardly be greater. Despite the fact that *in vitro* screening of traditional African antimalarial plants has met with little success, the screening process continues to be based on the same assumptions that have produced a series of commercial drugs with short effective lives. This raises a key question: If an indigenous plant with rapid parasite knockdown had been identified and had entered into widespread use at some point in Africa's past, would the therapy have been widely retained in the traditional pharmacopeia after resistance emerged? The absence of an African antimalarial compound with nanomolar antiplasmodial activity should therefore not be understood as a failure of the pharmacopeia but rather an indicator that the answer may lie in a different direction.

## 2. Outside-the-Box Epidemiological Contexts for Drug Development

Rather than supplying the next antiplasmodial wonder drug, African plants may play a more subtle and more interesting antimalarial role. Sub-Saharan Africa currently bears the heaviest burden of malaria worldwide. The presence of long-term human genetic adaptations to malaria in Africa, such as hemoglobinopathies and the Duffy-negative blood types, indicates that *Plasmodium* parasites have exerted genome changing selection pressure over millennia [28, 29]. There is evidence that human genetic adaptations to malaria in Africa may also include selection for tolerance of bitter dietary compounds with antimalarial activity [30, 31]. Related to this is the possibility that long-term exposure to malaria has driven African experimentation with plants and plant combinations that may alter the bloodstream environment in which *Plasmodium* parasites live. These more subtle and indirect processes may not be detectable in conventional *in vitro* screening of single plant extracts. Such plant-host-parasite interactions—discussed in more detail below—represent promising future research directions with potentially much higher rewards than merely finding another single molecule with nanomolar activity and a short clinical shelf life.

### 2.1. Acquired Immunity

Endemic malaria areas in sub-Saharan Africa are characterized by a high level of acquired “nonsterile” immunity in adults [32]. These adults typically have blood smears that are positive for *Plasmodium*, while their infections are largely asymptomatic. In order to understand how this resistance is acquired, a long-term study in Senegal compared the malaria epidemiologies of two villages with different transmission rates [33]. When the number of clinical malaria attacks in the villages was plotted against age, the trend indicated that there is an initial period of infant protection conferred via maternal immunity, followed by a rapid increase in severe malaria among young children, which subsequently declines later in childhood. The rate of acquisition of nonsterile immunity was faster in the village with the higher transmission rate, but was also clearly seen in the population with the lower transmission site.

 While the process of acquisition of malaria immunity reflects a natural immune system response to a high burden of malaria, it also raises the question of whether diet might influence immune parameters. The effects of dietary flavonoids on acquired immunity were modeled using *P. yoelii* in mouse trials—the results demonstrated that oral ingestion of a flavonoid-rich concentrate during initial infections significantly enhanced immune response to reinfection after treatment was discontinued [34]. In contrast, mice receiving pyrimethamine during the initial infection were vulnerable to severe reinfection when the drug was withdrawn, showing that drugs that completely eliminate parasites also stunt the development of immune memory. In the Senegal study, the introduction of standard-of-care antimalarial drugs eliminated malaria-related mortality but substantially increased the incidence of severe malaria [35]. Clearly there is an important long-term benefit to childhood exposure to parasitic infections, although acquisition of adult immunity may come at the cost of high infant mortality. Despite the prevalence of the phenomenon of acquired immunity in sub-Saharan Africa, the relationship between traditional plant therapies and immune processes has not been scientifically explored in humans in the field. If common dietary compounds such as flavonoids can influence the acquisition of immunity, further research could open up a promising new field in malaria therapy.

### 2.2. Diet and Bitter Tasting Compounds

Another possible role for diet in malaria epidemiology is hinted at in studies identifying human genetic traits distinctive to Africa. A potential link between malaria and diet was proposed in a study comparing the incidence malaria, frequency of sickle cell anemia and dietary intake of bitter cyanogenic glucosides present in cassava in Liberia [36]. An inverse relationship between the cyanide content of human diets and the frequency of hemoglobin S in four regions of Liberia suggested that cassava-derived cyanogenic glucosides might be replacing part of the antimalarial effects of the hemoglobin S. Sodium cyanate has been found to chemically modify hemoglobin S, increasing the survival and oxygen carrying capacity of sickle-cell erythrocytes [37]. Dietary cyanide levels corresponding to Liberian cassava intake were shown to significantly modify hemoglobin in a swine model, although it is not clear whether this should increase rather than decrease sickle cell prevalence [38].

 Several reports have noted an elevated frequency of distinctive alleles conferring a high threshold of bitterness detection in West African populations in high malaria areas [30, 31]. This tolerance of bitterness may be reflected in the wide popularity of cola nuts and the bitter tomato (*Solanum incanum* L.) and by regional preferences for fruits such as zegene (*Balanites aegyptiaca* L.), safou (*Dacryodes edulis* (G.Don) Lam), and ronier palm fruits (*Borassus aethiopum* Mart.) that are generally rejected by a European palate. The capacity to taste bitterness derives from specialized receptors on the tongue that are coded for by TAS2R genes, which are found in all vertebrates [39]. TAS2R genes are believed to be evolutionarily essential, acting as gateway sensors to bitter—and potentially toxic—compounds [39]. The low-sensitivity K172N allele, representing an earlier phylogenetic state, is found almost exclusively in tropical Africa but was subsequently replaced by the N172 mutant, which confers two-fold greater sensitivity to bitter taste [30]. This suggests that the evolutionary trade-off between avoidance of toxins and protection from malaria changed after human migration out of Africa [39]. Building on the earlier proposal of a cassava-malaria link, it was suggested that dietary cyanogenic glucosides may be the driver behind selection for reduced detection of bitter taste [30]. However, cassava is a relatively recent introduction from South America, while, according to phylogenetic reconstruction of the global dispersion of TAS2R alleles, high tolerance of bitter taste in Africa dates back at least 77,000 years [30, 40]. It is much more likely that other classes of bitter antimalarial compounds, such as dietary flavonoids with possible anticytoadherent and immunity-enhancing effects, would be the drivers for the putative selection for malaria-related bitterness tolerance.

 An additional line of evidence suggesting a strong role for diet in malaria tolerance comes from nonhuman primates. It has been observed that while forest monkeys are commonly infected with simian malarias, *Plasmodium* has never been seen in savanna monkeys in the wild, although captive monkeys have been experimentally infected with *P. gonderi* [41]. A study of Vervet and Patas monkeys in prime mosquito habitat along the Senegal River in the otherwise arid Ferlo region in northern Senegal failed to detect *Plasmodium*, haemosporidia, or microfilaria in blood smears sampled during peak mosquito periods [41]. Although the authors proposed that the lack of simian infections might be due to an absence of a mosquito vector specific to monkey malaria, this would not account for the failure to detect haemosporidia or microfilaria. There are several reasons to suspect that the intake of dietary compounds potentially relevant to malaria and other blood borne parasites could be substantially different between parasitized rainforest monkeys and unparasitized savanna monkeys. Savanna vegetation typically has a much higher content of many secondary metabolites than cooler or rainier areas [42]. Chemical analyses of plant samples taken along climate transects across the African savanna north of the equator show up to 15-fold higher tissue concentrations for some metabolites in the hottest and driest lowland savanna areas compared to cooler highlands [43, 44]. It is well documented that many plant secondary metabolites are increasingly synthesized under heat and drought stress, including flavonoids [45, 46]. Since savanna monkey diets include large quantities of flavonoid-rich leaves and bitter fruits such as *Balanites aegyptiaca*, it seems plausible that these dietary compounds could play a role in reducing or eliminating simian malaria. Computational analysis of the TAS2R gene family indicates that primates, including monkeys as well as humans, have experienced significant erosion in capacity to detect bitter taste compared to mice and a putative common ancestor, suggesting that an environment-driven evolutionary advantage is involved [47].

### 2.3. Plant Combinations

African antimalarial therapies often feature combinations of several plants. These mixtures may include analgesics and antipyretics as well as compounds that inhibit *Plasmodium* [11]. Of special interest is the possibility of chemically or physiologically complementary effects. Some alkaloids function as monoamine oxidase inhibitors (MAOis), which reduce the breakdown of active compounds by enzymes in the digestive tract, which in turn can result in a much higher uptake into blood plasma [48]. A well-known example of this from Amazonia is ayahuasca, a shamanic ceremonial and medicinal brew combining an MAOi donor plant containing harmane alkaloids with other plants containing visionary alkaloids, especially dimethyltryptamine [49]. While an equivalent traditional system has not been described in Africa, plants containing the necessary alkaloids are well represented in traditional pharmacopeias. Harmane alkaloids exhibit *in vitro* antiplasmodial activity, with a modest IC_50_ of 8 *μ*M reported for harmaline alone, although an additive effect of related compounds in the same plant may occur [50]. In North Africa, *Peganum harmala* L. has analgesic activity and is used to treat fevers and as an all-purpose medicinal plant [51]. Another harmane-containing plant, the savanna shrub *Guiera senegalensis* J.F.Gmel., is widely used as an antimalarial in the Sahel region [11]. *G. senegalensis* also contains guieranone, which is reported to have an *in vitro* antiplasmodial IC_50_ of 1.29 *μ*M [52]. Pure guieranone was, however, found to be cytotoxic towards monocytes and exhibited a low selectivity index, although the crude alkaloid extract showed low cytotoxicity [52]. Depending on the relative solubilities of harmane alkaloids and the more polar guieranone, traditional hot water extracts of *Guiera* may limit the amount of toxic material in the final preparation, which underscores the need for careful observation of traditional preparations [52]. A traditional combination of *Guiera* and *Mitragyna inermis* extracts showed strong *in vitro* synergism against the chloroquine-resistant W2 *P. falciparum* strain [52]. A closely related Southeast Asian tree, *M. speciosa*, contains over 25 alkaloids, including 7-hydroxymitragynine, which has an affinity for opioid receptors and exhibits more potent analgesic activity than morphine [53]. Although an MAOi effect has yet to be established in traditional African antimalarial therapies, some of the specific plant combinations employed suggest that this is very likely to be the case.

 Traditional African malaria therapies using combinations of plants may also affect active compound metabolism via interaction with cytochrome P450 (CYP), a large family of enzymes which account for about 75% of the chemical modification or degradation of drugs in humans [54]. As discussed earlier, African antimalarial plants often contain significant concentrations of flavonoids. Quercetin, a flavonoid found in many plants, has been reported to be an inhibitor of CYP2C9 and may in other cases function as an inducer of CYP3A4; although the processes involved are not well understood, quercetin can potentially alter the serum levels and the effects of drugs metabolized by these enzymes [55, 56]. Naringin, the principle flavonoid in citrus fruits, inhibits CYP3A4 and CYP1A2 and can affect drug absorption in the intestinal tract, potentially increasing or decreasing plasma drug concentrations [57]. Because consumption of normal dietary quantities of grapefruit or orange juice could affect circulating drug levels, citrus products may be included in drug interaction warnings [57]. Since traditional African combination therapies often involve flavonoid-rich species and plant parts, CYP interactions may enhance the plasma levels and activities of components of the preparation. Given thousands of years of exposure to malaria in Africa, the possibility that effective empirical combinations have been identified by traditional herbalists should be considered. There may be potential research dividends for closely observing traditional practices, especially where combination therapies using flavonoids with strong CYP activities are involved.

### 2.4. The Problem of Drug Targets

Although plant-based antimalarials such as quinine and artemisinin were already in ethnopharmaceutical use when first encountered by modern science, the current trend is to generate novel drug targets [21, 23]. Laboratories located far from the tropics—and consequently more in touch with technical aspects of the research and less aware of realities on the ground—have identified a multitude of physiologically essential metabolic pathways that can be targeted to kill parasites [23, 26]. In most cases, an effective new drug that emerges from this process and is deployed in the field can be expected to have a dismally short effective life before drug resistance develops, especially considering the years required for development, testing, and approval. The use of combination therapies and the rotation of drugs used in a given locality can slow or thwart the development of resistance; nevertheless, the threat of resistance always remains, with any lapse in the application of the drug administration strategy likely to be punished with a resurgence of severe malaria [35]. The emergence of drug resistance hinges on the application of direct selection pressure to the parasite. If a compound kills parasites by direct contact or exposure, then any genetic or physiological alteration that enhances survival will be retained. This means that virtually all parasite metabolic targets, no matter how ingenious, are destined to encounter resistance in the field, should the drug get that far in development.

 This is a fundamental problem at both conceptual and practical levels. However, the problem has an obvious solution: if long-term, sustainable efficacy is the goal, the therapy should primarily act upon the host rather than the parasite. This conclusion is not simply theoretical: it is abundantly attested to from the field, in the numerous human genetic adaptations to falciparum malaria. There is a growing body of evidence indicating that all of the malaria-related hemoglobin variants found in Africa result in a physiologically significant reduction in parasite cytoadhesion [58, 59]. *P. falciparum* must bind to endothelial cells lining the circulatory system to survive in the human host: parasites that do not cytoadhere will circulate into the spleen, where the infected red blood cells are detected and destroyed [60]. Thus, the high frequency of hemoglobinopathies in endemic malaria areas indicates that cytoadhesion is the evolutionary lever that has worked best against *P. falciparum* [58, 59]. What sets apart the disruption of cytoadhesion from other potential host responses to infection is the fact that no direct selection pressure to parasites is applied. The persistence of hemoglobinopathies over centuries demonstrates that malaria parasites have not evolved a viable defense against anticytoadherence. Following nature's lead, plant therapies that target cytoadhesion may stand a good chance of avoiding the drug-resistance cul-de-sac that limits the effective life of most antimalarial drugs.

### 2.5. Disruption of Parasite Cytoadhesion by Plant Compounds

Flavonoids, discussed earlier in their role as likely active compounds in many traditional African malaria therapies, have been shown to strongly affect the human endothelial system at normal dietary plasma concentrations, suggesting potential for reducing the severity of malaria infections and facilitating parasite clearance via immune response [34]. Endothelial cells expressing ICAM-1 proteins have been implicated as the principal binding sites for *P. falciparum*-infected erythrocytes in cerebral malaria [60]. ICAM-1 receptors in the brain microvasculature can anchor aggregates of infected erythrocytes, platelets, uninfected erythrocytes, and immune cells that block blood flow at multiple sites in the brain, often resulting in convulsions, coma and fatalities, most commonly in children [60]. ICAM proteins project out from blood vessel walls into the flow channel, sometimes shearing off to generate circulating soluble protein bodies that can exacerbate the blockage pathology [61]. A study reporting the effects of a cocoa concentrate beverage in patients at high risk for coronary heart disease found a mean increase of 76% in upper arm blood flow after six weeks of daily oral ingestion [61]. Levels of circulating soluble VCAM and lipids were significantly reduced. These effects are attributed primarily to epicatechin, a flavonoid found abundantly in cacao beans and cola nuts [61, 62]. Increases in plasma epicatechin also correlate to increases in plasma nitric oxide levels, which in turn increases vasodilation [61–63].

 A cocoa concentrate beverage tested on mice with *P. yoelii* infections reduced first challenge infections by as much as 50% although the specific mechanisms involved were not investigated [34]. Although cytoadhesion occurs in *P. yoelii*, all stages of erythrocytic parasites are commonly seen in peripheral blood, indicating that cytoadhesion is not as critical in rodent malaria as it is in human infections, with mice generally dying from hyperparasitemia. *In vitro* anticytoadhesion activity has been documented for epigallocatechin gallate (EGCG), a flavonoid that is commonly synthesized in plant leaves and is especially abundant in green tea [64]. EGCG was also found to alter the conformational structure of merozoite surface protein 1 [65]. Although merozoites do not cytoadhere, this finding suggests that it may be worth investigating the effect of this family of compounds on *P. falciparum* erythrocyte membrane proteins. Given the multiple endothelial and plasma environment altering effects of flavonoids, a significant disruption of *P. falciparum *infections is likely.

### 2.6. Plasma Concentration of Active Compounds

EGCG was found to competitively bind ICAM-1 *in vitro* at an IC_50_ of 5 *μ*M [64], which is just above the combined peak flavonoid concentrations in human plasma measured in green tea drinkers [66]. Since ECGC *in vivo* would be pulled out of circulation as it binds to ICAM receptors and plasma lipids, peak concentration is less critical than the total amount of drug in circulation, or area under the curve (AUC). It should be noted that for any drug targeting endothelial receptors, AUC is a much more relevant measure of competitive binding potential than peak plasma concentration. An additional consideration in evaluating flavonoids as anticytoadhesion drugs is that epicatechin and related compounds commonly occur as oligomers in plants [67]. Flavonoid oligomers have been shown to have greater *in vitro* nonspecific binding activity than monomer forms [68]. From this one might predict that polymerized flavanols will bind endothelial receptors at lower molar concentrations than monomers, thus lowering the plasma levels required for significant effect. Interestingly, the bitter taste of these compounds increases with the degree of polymerization, which may feed back into the prevalence of TAS2R alleles conferring low sensitivity to bitterness in human populations in endemic malaria areas of Africa.

 Polysaccharides constitute another class of polymerized plant compounds that are potentially anticytoadherent, although an investigation of carrageenans found that binding inhibition occurred only at physiologically impractical doses [69]. Africa has a large number of tree and shrub species that exude edible polysaccharide-based gums that are commonly employed in local cuisine and also enter medicinal use [11]. Since endothelial receptors are typically large proteins or glycoproteins, polysaccharides that are kilodaltons in size should theoretically be more efficient binders than small molecules, although they are much more likely to be enzymatically degraded as they pass through the digestive tract before entering blood plasma [69, 70].

 In conventional thinking, a good antimalarial plant is one that possesses an active compound that inhibits *Plasmodium* at a few parts per billion, well below the potential plasma concentration of the orally ingested compound [15, 17, 20]. In contrast, most antimalarial compounds identified in African pharmacopeias have relatively weak parasite killing activity, with *in vitro* IC_50_ values in parts per million [12–16]. Since blood plasma levels of presumed active compounds in most African antimalarial plants are commonly below the concentration thresholds for direct antiparasite activity, the logical conclusion is that the plants are marginally effective at best, unless other mechanisms are involved. Simply not enough compound is present to directly inhibit malaria parasites.

 This raises the question of why a plant lacking a compound with nanomolar antiparasitic activity enters into the local antimalarial pharmacopeia in the first place. While the explanation may be that the compounds are simply treating fever or inflammation, this commentary makes a case for multiple mechanisms that can potentially change the concentration requirements for active compounds. These may include influencing immune memory acquisition, inhibiting or inducing enzymatic processes affecting drug metabolism, competitive binding of endothelial receptors, increasing nitric oxide levels, disaggregation of platelets, and vasodilation [34, 61–64]. The most important point with regard to active compound concentration is that direct parasiticidal effects may not be the goal of traditional African antimalarial therapy. In fact, from the perspective of maintaining a sustainable, viable indigenous therapy over centuries, direct elimination of parasites is self defeating.

## 3. Conclusion

The potential contribution of African plants to mainstream malaria therapy may take the form of alternative strategies for treating the disease. Clues from human genetic adaptations to malaria suggest that cytoadhesion may be the key target. *P. falciparum* must cytoadhere in order to survive in a human host. More importantly, while cytoadhesion is essential to the parasite, interference with cytoadhesion does not apply direct selection for resistance. It is therefore not a coincidence that the suite of hemoglobinopathies so far discovered in Africa reduce the endothelial binding capacity of parasites [58, 59]. The prevalence of host anticytoadhesion traits in African populations strongly impacted by malaria represents a long-term evolutionary strategy that can inform malaria drug development. To date, the potential role of plants found in African antimalarial pharmacopeias against cytoadhesion has been largely overlooked.

 The historical role of diet as a significant component in the African experience with malaria has also been overlooked, yet it is strongly hinted at by the prevalence of low human sensitivity to bitter taste in endemic malaria areas. Bitter plants in African diets contain several families of compounds with known antimalarial activity. Despite very limited research carried out so far, the potential of flavonoids is especially intriguing since these compounds exhibit a suite of physiological effects that may counteract cytoadhesion and enhance immune response to malaria.

 One theme running through all of these alternative mechanisms for malaria therapy is that *in vitro* assays are very unlikely to reveal any relevant compound activity. Screening plants for *in vitro* nanomolar IC_50_ values is the standard approach in plant-based antimalarial research. This approach unthinkingly assumes that direct control of parasites is the only viable goal. However, in every known case of an effective plasmodicidal drug entering wide use, parasites have developed resistance [33].

 What is an ideal antimalarial drug? An ideal drug is one that is sustainable, maintaining efficacy over long-term field use. Contrary to the assumption in most antimalarial compound screening, *in vitro* tests demonstrating low-dose antiplasmodial activity may instead serve to flag the compound as a probable driver of resistance against itself. This argument does not deny the important role in malaria treatment played by drugs that effectively kill parasites at low doses—these are very much needed for treatment of clinical malaria. The case is simply being made that long-term, community-wide use of drugs that apply direct selection pressure to parasites will always face the prospect of emerging resistance. To get beyond drugs with limited effective life spans, a much greater emphasis on *in vivo* testing is required to capture the interaction between host, parasite, and drug. While *in vivo* research can be much more laborious with far lower throughput compared to *in vitro* screening, the host-centered process may well yield better drugs. In the case of many antimalarial plants and herbal combination therapies widely used in African pharmacopeias, *in vivo *testing may represent the only means of recognizing and measuring the efficacy of the traditional therapy.
